# Electroporation and in vitro culture of early rabbit embryos

**DOI:** 10.1016/j.dib.2018.09.131

**Published:** 2018-10-04

**Authors:** Erin Betters, Rebekah M. Charney, Martín I. García-Castro

**Affiliations:** School of Medicine Division of Biomedical Sciences, University of California, Riverside, CA 92521, USA

## Abstract

The functional interrogation of factors underlying early mammalian development is necessary for the understanding and amelioration of human health conditions. The associated article [1] reports on the molecular characterization of markers of neural crest cells in gastrula and neurula stage rabbit embryos. This article presents survival data of rabbit embryos cultured in vitro, as well as immunofluorescence data for molecular markers of neural crest cells following approximately 24-h of culture. Lastly, towards the functional analysis of early neural crest and other developmental genes, this article provides data on the introduction of exogenous DNA into early stage rabbit embryos using electroporation.

**Specifications table**

**Table:** 

Subject area	*Biology*
More specific subject area	*Developmental Biology*
Type of data	*Table, images*
How data was acquired	*Images were acquired using a Spot camera on a Nikon Eclipse 80i.*
Data format	*Raw, processed images*
Experimental factors	*Rabbit embryos were collected from timed-pregnant New Zealand White rabbits.*
Experimental features	*Wild-type rabbit embryos were cultured in vitro for 24-h and assayed for morphology and molecular markers of neural crest cells. Embryos were injected with morpholino oligonucleotides (lis-MO) and/or plasmid DNA, electroporated, and cultured overnight.*
Data source location	*School of Medicine Division of Biomedical Sciences, University of California, Riverside U.S.A.*
Data accessibility	*Data is within this article*
Related research article	*Betters, E., Charney, R.M., Garcia-Castro, M.I. Early specification and development of rabbit neural crest cells. Developmental Biology. In press.*

**Value of the data**

• This data demonstrates the first successful incorporation of exogenous DNA into rabbit embryos using electroporation.
• Embryo survival data and marker gene expression following 24-h culture at multiple stages will be relevant to inform future experiments using the rabbit model system.
• Towards efforts to examine the functional role of early developmental genes in the rabbit, these data together provide important experimental guidance on embryological manipulations using the rabbit embryo.

## 1 Data

We present data on the survival rates of in vitro cultured gastrula and neurula stage rabbit embryos, and use immunofluorescence to stain cultured rabbit embryos for molecular markers of neural crest cells, including Pax3, Pax7, and Sox10. We also provide data on the cellular introduction of exogenous DNA (morpholino oligonucleotides and plasmids) into rabbit embryos using electroporation, and the successful culture thereafter.

## 2 Experimental design, materials, and methods

### 2.1 Embryo collection and staging

Timed-pregnant New Zealand White rabbits (Millbrook Breeding Labs, Amherst, MA) were euthanized, and uteri removed and stored in 1x PBS at 4 °C. Embryos were dissected from the uterine tissue in sterile 1x PBS containing 5% Fetal Bovine Serum (FBS) and 100 U/mL Penicillin-Streptomycin (Thermo Fisher). Embryos were staged as previously reported, with pre-gastrula embryos according to Viebahn et al. [2], [3], and gastrula and neurula embryos according to chick developmental stages [3], [4]. Rabbit embryos were obtained and experiments performed at Yale University, following the approval and guidelines of IACUC.

### 2.2 in vitro culture of rabbit embryos

Whole rabbit embryos between St. 1 and 3 somite stage were cultured using the “ring method” as previously described [5] at 37 °C with 5% CO2 for 19–22. Following culture, embryos were cryosectioned and processed for immunofluorescence as described below. Successful cultures were marked as those embryos demonstrating normal morphology along the entire rostral-caudal axis (Table 1).

**Table 1:** *Survival rates for the in vitro culture of rabbit embryos*. All embryos were cultured using the “ring culture” method as described by Püschel et al. [5]. Rabbit embryos can be cultured from St. 1 to St. 2 with a high degree of success (67%); however, in our hands, the success rate drops dramatically when embryos between St. 3 and St. 4 are placed into culture (17%). Following gastrulation stages, rabbit embryos at St. 5 and older can be cultured up to the 7–8ss, with embryos generally demonstrating normal morphology (i.e. neural folds/neural tube and somites) (Fig. 1C-D). The success rate for these older cultures hovers between 67–83%, depending on the starting embryonic stage. We also note the successful culture of a St. 3 embryo cultured to the St. 7 cranial stage (following 48-h culture), although this embryo was deformed caudally and not included in the table. Although the successful culture of rabbit embryos as young as St. 4 through the early somite stages has been reported, these embryos often display abnormal marker gene expression [7].

**Pre-Culture (stage)**	**Post-Culture (stage)**	**Culture Time (h)**	**Surviving Embryos**	**Percentage (%)**
St. 1	St. 2	19–22	4/6	67%
St. 3–4	St. 5	19–22	1/6	17%
St. 4+/5	St. 6 to 5ss	19–22	5/6	83%
St. 6 to 3ss	3ss to 8ss	19–22	8/13	62%

### 2.3 Immunohistochemistry

For immunohistochemistry, rabbit embryos were fixed in 4% paraformaldehyde for 30 min to 1.5 h (depending on the stage) at room temperature. Embryos were embedded in gelatin and cryosectioned (Leica CM1900), or subjected to whole mount immunofluorescence. Sections were stained for markers of NC including AP2α, Msx1/2, Pax3, Pax7, Sox9, and Sox10, and the neural markers Sox2/3, as described in the associated article [1].

### 2.4 Electroporation of rabbit embryos

Embryos (St. 4+ to St. 6 and 3–4ss) intended for electroporations were prepared for whole mount culture as above. Following the removal of excess culture media, embryos were injected with a mixture of the following: Sucrose (1.7–2%), non-targeting Lissamine-morpholino oligonucleotide (0.33–0.4 mM), pCIG (1ug/uL), and Fast Green. Injections typically targeted the right side of the embryonic disc. Electroporations were performed with 5.5–6 V (n=4; St. 4+/5) or 6.5 V (n=7; St. 5/6 to 3–4ss) with 5 pulses of 50 ms “On,” 100 ms “Off.” Rings were placed over the extraembryonic tissue framing the embryos post-electroporation, and subsequently filled with culture media. Electroporated embryos were cultured overnight. All embryos electroporated at 5.5–6 V demonstrated normal morphology post-culture, and morpholino signal was observed in all examples (n=4/4). All electroporations were performed with a dual-pronged rod, as has previously been described for the in vivo electroporation of chick embryos [6]. We also note that all embryos electroporated with 6.5 V demonstrated abnormal morphology post-culture, with caudal defects typically observed. Definitive neural fold development was observed rostrally in few examples (n=2/7), with these embryos retaining morpholino expression on the side of electroporation (Fig. 2, Fig. 3).

**Fig. 1:**
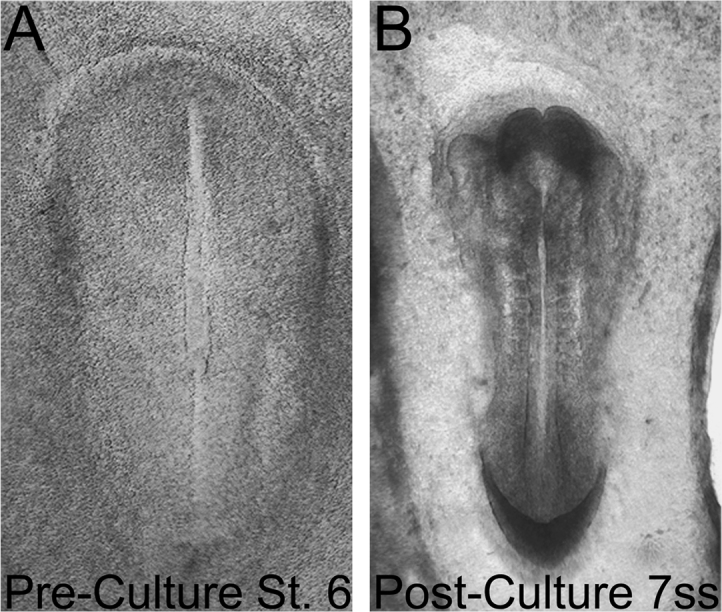
*Morphology of in vitro cultured rabbit embryo*. Example of a rabbit embryo developed in vitro from (A) St. 6 to (B) the 7ss. After culture, the 7ss embryos displays normal morphology including formation of neural folds, somites, and heart primordia.

**Fig. 2:**
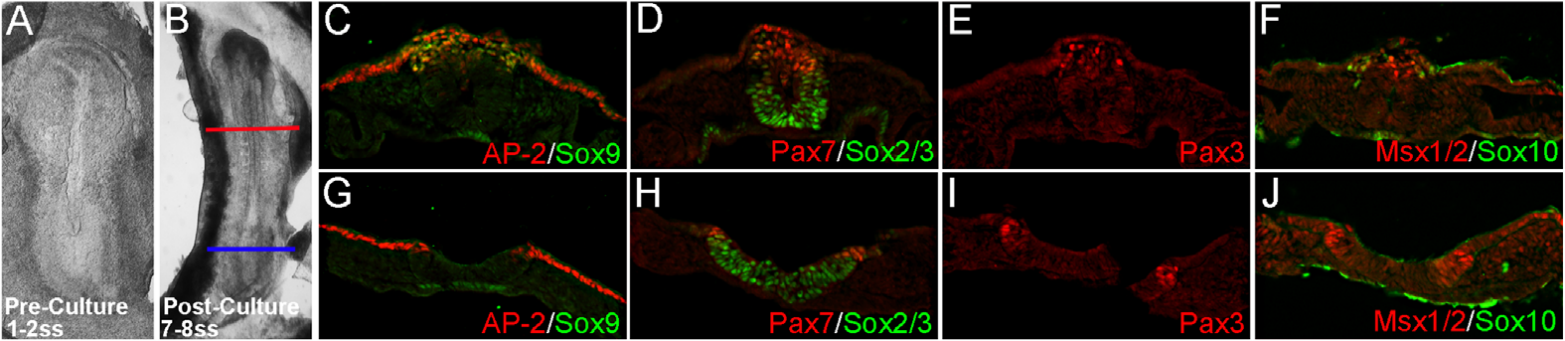
*Cultured rabbit embryos express characteristic molecular markers of neural crest cells*. (A) 1–2ss embryo was cultured for 24-h to (B) 7–8ss. (C-J) Cryosections taken from axial levels marked in (B) and immunostained for neural crest markers. Red and blue lines indicate the axial levels of sections in (C-F) and (G-J), respectively. These data reveal patterns of neural crest gene expression from an example of an in vitro cultured embryo that is largely consistent with those observed *in vivo*[1]. Similar data was obtained from all embryos assayed (n=7/7). (C-F) AP2α, Sox9, Pax3, and Sox10 are all observed in presumptive migratory NC cells directly above and/or dorsolateral to the newly formed neural tube. We note a small number of Pax7-positive migratory NC cells, but the majority of signal is found in the pre-migratory NC found in the dorsal aspect of the neural tube. Similarly, Msx1/2 is predominantly expressed in pre-migratory crest cells. (G-J) AP2α, Pax7, Pax3 and Msx1/2 signal are observed in pre-migratory NC cells within the more caudal neural folds. Sox9 and Sox10 expression is not observed. (D, H) Sox2/3 staining denotes the neuroepithelium.

**Fig. 3:**
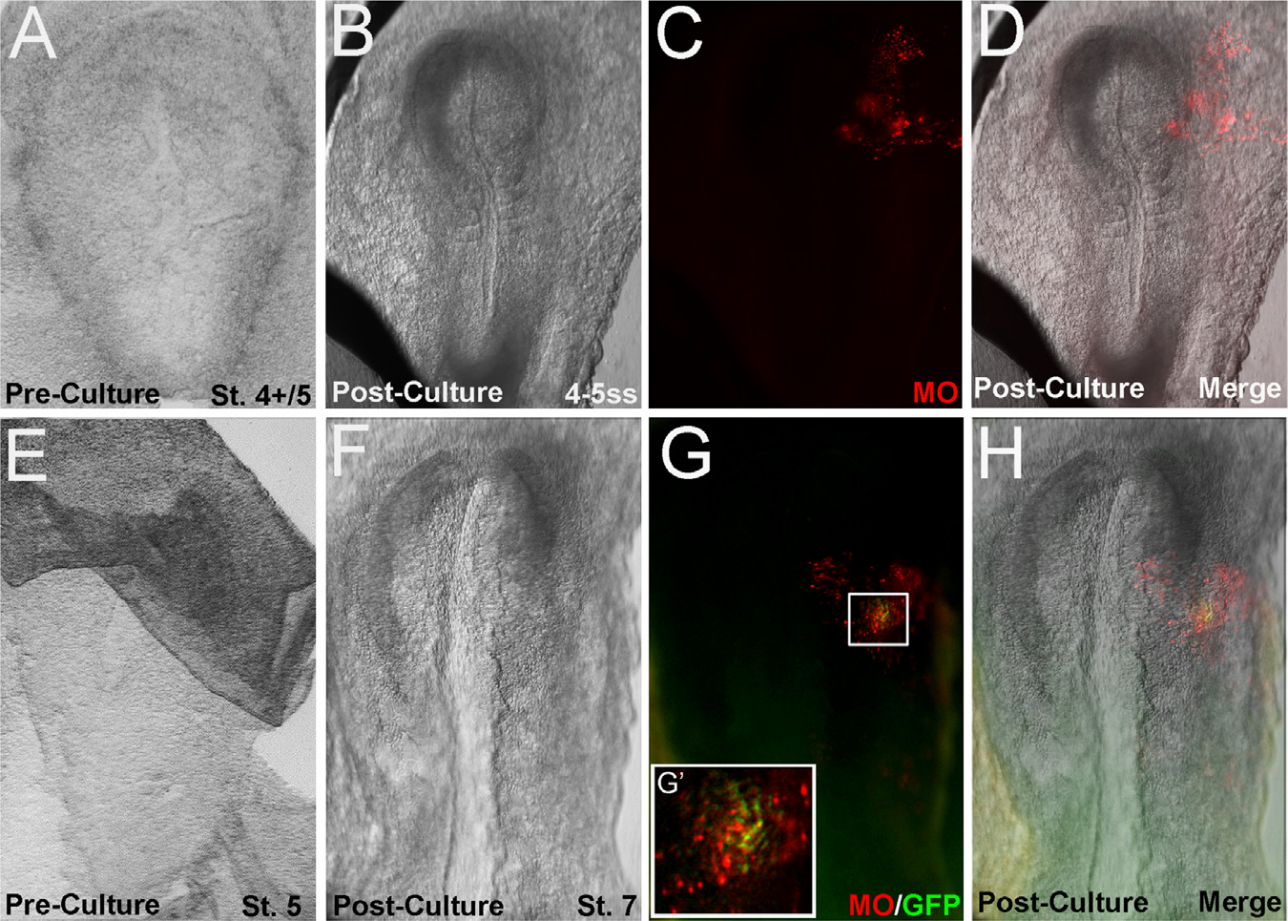
*Electroporation of exogenous DNA into rabbit embryos.* Two examples of rabbit embryos electroporated with either non-targeting morpholino oligonucleotide (lissamine-MO) alone (A-D) or in conjunction with pCIG (GFP) plasmid (E-H) and cultured overnight. In all cases (n=4/4), embryos electroporated with lissamine-MO demonstrate fluorescence post-culture (C, D, G, H), indicating MO incorporation in the cells. We also observed a small number of GFP-expressing cells in embryos co-injected with lissamine-MO and pCIG (G, H) (n=2/4). However, we note that GFP expression was observed n=2/4 embryos tested.
